# Abemaciclib plus endocrine therapy versus chemotherapy after progression on prior palbociclib in HR+/HER2‐ metastatic breast cancer: A single center real‐world study in China

**DOI:** 10.1002/cam4.7249

**Published:** 2024-05-21

**Authors:** Hanfang Jiang, Jianxin Zhong, Jing Wang, Guohong Song, Lijun Di, Bin Shao, Ruyan Zhang, Yaxin Liu, Anjie Zhu, Nan Wang, Huiping Li

**Affiliations:** ^1^ Key Laboratory of Carcinogenesis and Translational Research (Ministry of Education), Department of Breast Oncology Peking University Cancer Hospital & Institute Beijing China

**Keywords:** abemaciclib, chemotherapy, endocrine therapy, hormone receptor‐positive, metastatic breast cancer, palbociclib progression

## Abstract

**Background:**

Cyclin‐dependent kinase (CDK) 4/6 inhibitor plus endocrine therapy (ET) become standard‐of‐care for patients with hormone receptor‐positive, human epidermal growth factor receptor‐2 negative (HR+/HER2−) metastatic breast cancer (MBC). However, the optimal therapeutic paradigm after progression on CDK4/6 inhibitor remains unclear. This study aimed to evaluate the efficacy and safety of abemaciclib with switching ET versus chemotherapy after progression on prior palbociclib‐based ET in Chinese patients with HR+/HER2− MBC.

**Methods:**

From 414 consecutive patients with HR+/HER2− MBC who had been treated with palbociclib plus ET from September 2018 to May 2022 in Peking University Cancer Hospital, we identified 80 patients who received abemaciclib plus switching ET or chemotherapy after progression on palbociclib, matched for age, original stage at diagnosis, disease‐free interval, and tumor burden at 1:1 ratio. The primary endpoint was progression‐free survival (PFS) compared using the Kaplan–Meier method. A Cox proportional hazard model was performed to identify clinical factors associated with PFS in the abemaciclib group.

**Results:**

The median PFS was 6.0 months (95% confidence interval [CI]: 3.94–8.06) in abemaciclib group and 4.0 months (95% CI, 2.52–5.49) in chemotherapy group (*p* = 0.667). And, there was no difference in median PFS between the sequential and nonsequential arm (6.0 vs. 6.0 months) in the abemaciclib group though fewer lines of prior systemic therapy and longer PFS from prior palbociclib in the sequential arm. However, patients with prior palbociclib as the first‐line therapy had a significantly longer median PFS versus prior palbociclib as ≥2nd‐line therapy (11.0 vs. 5.0 months, *p* = 0.043). Based on multivariable analysis, ER+/PR+ was an independent factor associated with longer PFS. There was no significant difference in overall survival between the abemaciclib and chemotherapy groups (*p* = 0.069).

**Conclusion:**

Our findings indicate that abemaciclib plus switching ET might be one of feasible treatment options for Chinese patients with HR+/HER2− MBC after progression on prior palbociclib‐based therapy in addition to chemotherapy.

## 1 INTRODUCTION

Breast cancer (BC) is the most frequently diagnosed malignancy and the leading cause of cancer‐related mortality in women worldwide.^1^ Thereinto, hormone receptor (HR)‐positive, human epidermal growth factor receptor‐2 (HER2) negative (HR+/HER2‐) tumor is the most common subtype, accounting for approximately 75% of all BC cases.^2^ Over the past approximately five decades, endocrine therapy (ET) had been the standard front‐line treatment for patients with HR+/HER2‐ metastatic breast cancer (MBC).^3^ However, most patients will ultimately experience resistance to ET and disease progression. HR+/HER2‐ metastatic MBC remains incurable, with a median overall survival (OS) of approximately 4–5 years.^4^, ^5^

Cyclin‐dependent kinases (CDK) 4/6 play a pivotal role in regulating the transition from the G1 stage to the S stage of the cell cycle.^6^ The dysregulation of the CDK 4/6 pathway is one of the most common mechanisms associated with breast carcinogenesis and ET resistance in HR+/HER2‐ MBC.^7^, ^8^ In the last decade, several CDK4/6 inhibitors have been developed to overcome ET resistance. The pivotal phase Ш PALOMA‐2, −3,^9^, ^10^, ^11^ MONARCH‐2, −3,^12^, ^13^, ^14^ MONALEESA‐7, −2, −3,^15^, ^16^, ^17^ and DAWNA‐1, −2^18^, ^19^ clinical trials all demonstrated that CDK4/6 inhibitors in combination with ET have significantly improved progression‐free survival (PFS) compared with ET alone. Notably, update data from MONARCH‐2,^20^ MONALEESA‐7,^21^ MONALEES‐3,^22^ and MONALEESA‐2^23^ have also shown abemaciclib or ribociclib plus ET had significantly prolonger OS compared to ET alone. Currently, CDK4/6 inhibitor in combination with ET become the new standard‐of‐care in the first‐ or second‐line settings for patients with HR+/HER2‐ MBC.^24^, ^25^, ^26^, ^27^

So far, there have been three CDK4/6 inhibitors successively approved by the United States Food and Drug Administration (FDA) for the treatment of HR+/HER2‐ advanced or MBC in combination with an aromatase inhibitor or fulvestrant as initial endocrine‐based therapy or after disease progression following ET: palbociclib as the first CDK4/6 inhibitor approved in February 2015, then ribociclib approved in March 2017, and abemaciclib approved in September 2017.^28^ Whereas, there have been four CDK4/6 inhibitors successively approved by National Medical Products Administration in China: palbociclib was approved in July 2018, then abemaciclib was approved in December 2020, dalpiciclib approved in December 2021, and ribociclib was approved in January 2023. However, despite the clinical benefit from these agents being striking, patients will still eventually develop drug resistance.^29^ The optimal therapeutic paradigm after progression on CDK4/6 inhibitor has emerged as an unsolved medical problem.

Abemaciclib is the only CDK 4 / 6 inhibitor approved by FDA as a monotherapy for patients with HR+/HER2‐ MBC heavily pretreated with both ET and chemotherapy, based on the results from MONARCH‐1.^30^ Furthermore, abemaciclib was proven to cross the blood‐brain barrier and have distinct activity in brain metastases.^31^, ^32^ In a preclinical study, abemaciclib was demonstrated to have activity in palbociclib‐resistant and ‐adapted BC cells.^33^ There is increasing interest in continuing abemaciclib after progression on palbociclib in clinical practice.^34^ In our center, some clinicians have administered abemaciclib plus ET in patients with HR+/HER2‐ MBC who had received palbociclib‐based therapy and experienced disease progression, mostly due to patients' refusal or intolerance of chemotherapy in the clinical practice since 2021. Nevertheless, data about subsequent abemaciclib in combination with ET after palbociclib progression is still limited.

Thus, this single‐center retrospective study aimed to evaluate the efficacy and safety of abemaciclib in combination with switching ET versus chemotherapy after progression on prior palbociclib‐based ET in patients with HR+/HER2‐ MBC in a real‐world setting in China.

## 2 PATIENTS AND METHODS

### 2.1 Study design

This was a single‐center, retrospective, observational study evaluating the efficacy and safety of abemaciclib in combination with ET versus chemotherapy after progression on prior palbociclib‐based ET in Chinese patients with HR+/HER2‐ MBC in a real‐world setting at Peking University Cancer Hospital. This study was approved by the Ethics Committee of Peking University Cancer Hospital (Approval ID: 2022KT27), which waived the requirement for patient‐signed informed consent owing to the retrospective nature of the study.

### 2.2 Patients

All consecutive patients diagnosed with pathologically confirmed HR+/HER2‐ MBC who had been treated with palbociclib were reviewed via the electronic medical record system at Peking University Cancer Hospital from September 2018 to May 2022. The inclusion criteria for the case group were as follows: (1) female patients aged ≥18 years old with pathological confirmed HR+/HER2‐ MBC; (2) disease progression on previous palbociclib in combination with ET in the metastatic setting; (3) received abemaciclib plus switching ET treatment afterward; (4) Eastern Cooperative Oncology Group performance status (ECOG PS) of 0–2 at initiating abemaciclib plus ET; and (5) at least one measurable lesion according to Response Evaluation Criteria in Solid Tumors version 1.1 (RECIST v1.1)^35^ at initiating abemaciclib plus ET. The exclusion criteria included discontinued prior palbociclib due to adverse effects, palbociclib switched to abemaciclib due to an adjusted medical insurance policy in which abemaciclib has been covered by the medical insurance system since January 2022 in China, receiving abemaciclib for less than 30 days, continuing the same ET, evidence of another primary cancer within 5 years, or incomplete medical information in the electric medical record system.

The chemotherapy group was selected from those patients who received chemotherapy after progression on palbociclib‐based therapy. We matched one patient who received chemotherapy to each patient who received abemaciclib plus ET by age (plus/minus 2 years) at the start of abemaciclib or chemotherapy, original stage at diagnosis, disease‐free interval (DFI), and site of metastatic lesion (visceral or nonvisceral) and number of metastatic sites at the start of therapy. We applied the same inclusion and exclusion criteria to the chemotherapy group as to the abemaciclib group except for receiving abemaciclib in combination with endocrine therapy.

### 2.3 Treatment and data collection

In the abemaciclib group, abemaciclib was administered orally twice a day, combined with ET. Premenopausal patients who received an AI or fulvestrant were concomitantly administered with ovarian function suppression and patients received chemotherapy of physician's choice in the control group. All eligible patients' demographics and baseline pathological characteristics including patient age, menopausal status at the start of abemaciclib or chemotherapy, disease‐free survival, the latest pathological result from either primary tumor or metastatic lesion, previous treatment history in the metastatic setting including ET and chemotherapy regimen, number and type of metastatic sites at the initiation of abemaciclib, ECOG PS at the start of abemaciclib or chemotherapy were collected. And, the regimen of abemaciclib combined ET or chemotherapy and the clinical outcomes including the start date of abemaciclib or chemotherapy, the initial dose of abemaciclib or chemotherapy, type of ET or chemotherapy, efficacy and safety of abemaciclib plus ET or chemotherapy, cause of dose adjustments, and the date of disease progression were obtained from the electronic medical record system through reviewing the patient's medical records. Aromatase inhibitors (AIs) included letrozole, anastrozole, or exemestane. Survival status was collected from the medical record system or by making telephone call follow‐ups. The follow‐up interval was every 2–3 months for abemaciclib or every 2–3 cycles for chemotherapy. Data collection was cut off on April 30th, 2023, the date of death, or date of last follow‐up (if lost to follow‐up), whichever came first.

HR‐positive was defined as a minimum of 1% of invasive tumor cells positive for estrogen receptor (ER) and/or progesterone receptor (PgR) by immunohistochemistry (IHC).^36^ HER2‐ negative was defined as IHC score of 0 or 1+ or IHC score of 2 with fluorescence in‐situ hybridization (FISH) negative.^37^

### 2.4 Study endpoints

The primary endpoint of our study was progression‐free survival (PFS). The secondary endpoints included objective response rate (ORR), disease control rate (DCR), clinical benefit rate (CBR), OS, and safety. PFS was calculated as the start of abemaciclib in combination with ET or chemotherapy to disease progression or the date of death from any cause without progression, whichever came first. OS was calculated as the time from the date of abemaciclib or chemotherapy initiation to death of any cause or last follow‐up. The response was evaluated per RECIST version 1.1^35^ based on computerized tomography (CT) scan or magnetic resonance imaging (MRI), and the medical documentation of the treating clinicians' assessment. ORR was defined as the proportion of patients with complete response (CR) or partial response (PR) as the best overall response. DCR was defined as the proportion of CR, PR, or stable disease (SD), and CBR was defined as CR + PR + SD ≥6 months. The incidence and severity of adverse events (AEs) were recorded from the treating clinicians' documentation and blood analyses according to the Common Terminology Criteria for Adverse Events (CTCAE) version 5.0.^38^

### 2.5 Statistical analyses

Demographics and clinical characteristics were summarized using descriptive statistics. Efficacy analyses were performed for patients who had at least one assessment of tumor response. Differences between groups were compared by Pearson's chi‐squared test or Fisher's exact test where appropriate for categorical variables, and by Student *t* test or Mann–Whitney test for continuous variables. PFS and OS were estimated using the Kaplan–Meier method, along with median estimates with 95% confidence intervals (CI), and subgroups were compared using a log‐rank test. A Cox proportional hazard model was used to estimate hazard ratios (HRs) with the corresponding 95% CIs of variables influencing PFS. All variables with a *p*‐value <0.1 in the univariate Cox analyses were included in the multivariate analysis.

All statistical analyses were performed using SPSS software version 27.0 (SPSS, Inc., Chicago, IL). All tests were two‐sided and statistical significance was defined as *p* < 0.05.

## 3 RESULTS

### 3.1 Patients' demographics and baseline characteristics

**FIGURE 1:**
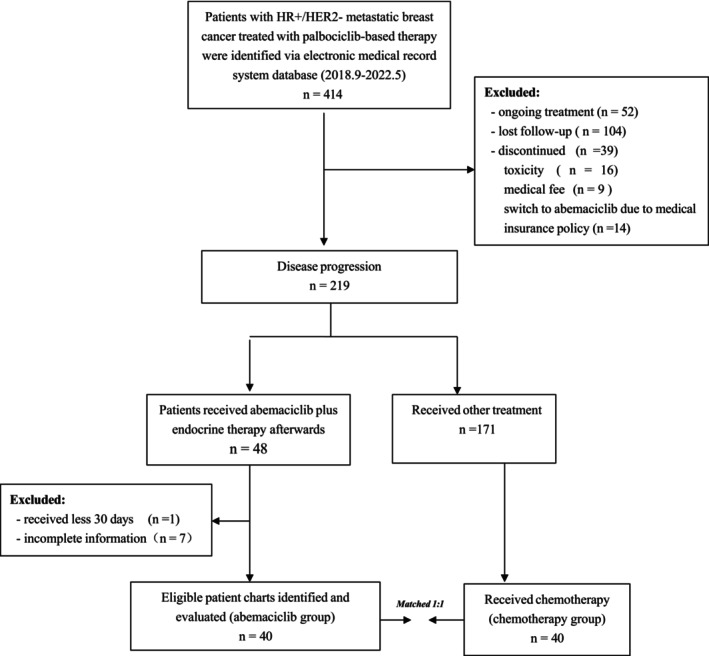
Flow diagram of patient selection and study design. HR, hormone receptors; HER2, human epidermal growth factor receptor‐2.

Between September 2018 and May 2022, a total of 414 patients with HR+/HER2‐ mBC were identified using the electronic medical record system who received palbociclib in combination with ET in the metastatic setting at Peking University Cancer Hospital. Then, 219 patients had disease progression and only 48 patients were identified to receive abemaciclib plus ET afterward, while 171 patients received other treatment afterward. After eight patients were excluded (one patient due to discontinued abemaciclib less than <30 days, and seven patients due to lack of complete medical information), a total of 40 patients were included in the abemaciclib group of the study. Another 40 patients who received chemotherapy of physician's choice afterward were matched to each patient in the abemaciclib group of the study (Figure 1). In total, 80 patients were included in the study.

Patient demographics and baseline clinical characteristics are shown in Table 1. Overall, the median age at the start of therapy was 60.0 years (range: 36–84 years) in the abemaciclib group and 59.5 years (range: 35–86) in the chemotherapy group.

**TABLE 1:** Patient demographics and clinical characteristics of the abemaciclib group and chemotherapy group at baseline.

Characteristic	Abemaciclib group (*n* = 40) *n* (%)	Chemotherapy group (*n* = 40) *n* (%)	*p* value
Female	40 (100.0)	40 (100.0)	
Age at diagnosis of breast cancer, years			
Mean ± SD	49.9 ± 11.6	50.7 ± 11.1	0.768
Median (range)	49.0 (30–73)	50.0 (28–71)	
TNM stage at diagnosis			1.000
I/II	17 (42.5)	17 (42.5)	
III	10 (25.0)	10 (25.0)	
IV	13 (32.5)	13 (32.5)	
Disease‐free interval			1.000
De novo	13 (32.5)	13 (32.5)	
≤2 years	2 (5.0)	2 (5.0)	
>2 years	25 (62.5)	25 (62.5)	
Age at the start of abemaciclib or chemotherapy, years			
Mean ± SD	59.5 ± 12.7	58.4 ± 12.4	0.716
Median age (range)	60.0 (36–84)	59.5 (35–86)	
Age group			0.588
<50 years	10 (25.0)	7 (17.5)	
50–64 years	14 (35.0)	18 (45.0)	
≥ 65 years	16 (40.0)	15 (37.5)	
Menopausal status at the start of therapy			0.626
Premenopausal	13 (32.5)	11 (27.5)	
Postmenopausal	27 (67.5)	29 (72.5)	
ECOG PS status at the start of therapy			0.592
0	30 (75.0)	32 (80.0)	
1	10 (25.0)	8 (20.0)	
Histological type			0.263
Invasive ductal	34 (85.0)	38 (95.0)	
Others	6 (15.0)	2 (5.0)	
Hormone receptor status			0.412
ER positive/PgR positive	30(75.0)	33 (82.5)	
ER positive/PgR negative	10 (25.0)	7 (17.5)	
Metastatic site at the start of therapy			
Bone	33 (82.5)	32 (80.0)	0.775
Lymph nodes/soft tissues	25 (62.5)	28(70.0)	0.805
Lung	22 (55.0)	23 (57.5)	1.000
Liver	18 (45.0)	25 (62.5)	0.116
Brain	4 (10.0)	3 (7.5)	1.000
Site of metastatic lesion at the start of therapy			0.576
Visceral	31 (77.5)	33 (82.5)	
Nonvisceral	9 (22.5)	7 (17.5)	
Number of metastatic sites at start of therapy			1.000
1	3 (7.5)	3 (7.5)	
2	8 (20.0)	8 (20.0)	
≥3	29 (72.5)	29 (72.5)	
Number of prior lines in the metastatic setting, median (range)			
Endocrine	2 (1–6)	2 (1–7)	0.178
Chemotherapy	1 (0–12)	0 (0–6)	0.011^a^
Total	3 (1–18)	2 (1–10)	0.026^a^
Line of prior endocrine therapy in the metastatic setting			0.340
1	11 (27.5)	15 (37.5)	
≥2	29 (72.5)	25 (62.5)	
Line of prior chemotherapy in the metastatic setting			0.058
0	12 (30.0)	21 (52.5)	
1	11 (27.5)	11 (27.5)	
≥2	17 (42.5)	8 (20.00	
Line of prior palbociclib‐based therapy			0.302
1st	8 (20.0)	12 (30.0)	
≥ 2nd	32 (80.0)	28 (70.0)	
Prior endocrine therapy combined with palbociclib			0.037^a^
Aromatase inhibitor	22 (55.0)	20 (50.0)	
Fulvestrant	13 (32.5)	20 (50.0)	
Tamoxifen/toremifene	5 (12.5)	0	
Initial dose of Palbociclib			0.098
125 mg	21 (52.5)	30 (75.0)	
100 mg	15 (37.5)	7 (17.5)	
75 mg	4 (10.0)	3 (7.5)	
Best response of prior endocrine therapy plus palbociclib			
ORR (CR + PR)	12 (30.0)	2 (5.0)	0.003^a^
DCR (CR + PR + SD)	35 (87.5)	30 (75.0)	0.152
Median duration of prior palbociclib‐based therapy (range), months	10 (2–24)	7 (1–20)	0.014^a^
1st	14.0 (9.8–18.2)	7.0 (3.9–10.1)	0.121
≥2nd	9.0 (7.2–10.8)	7.0 (1.9–12.1)	0.045^a^
PFS of prior palbociclib‐based therapy			0.036^a^
<12 months	21 (52.5)	30 (75.0)	
≥12 months	19 (47.5)	10 (25.0)	
CDK4/6 inhibitor sequence			
Sequential	23 (57.5)		
Nonsequential	17 (42.5)		
Number of prior systemic therapy lines at start of abemaciclib, median (range)			<0.001^a^
Sequential	2 (1–7)		
Nonsequential	6 (3–18)		
PFS of prior palbociclib in Abemaciclib group, median (range), months			0.021^a^
Sequential	12.0 (4.17–19.8)		
Nonsequential	10.0 (5.17–14.83)		
Abemaciclib‐based regimen	40 (100)		
Aromatase inhibitor	16 (40.0)		
Fulvestrant	18 (45.0)		
Megestrol Acetate	6 (15.0)		
Concomitant OFS	13 (32.5)		
Initial dose of abemaciclib			
150 mg	18 (45.0)		
100 mg	17 (42.5)		
50 mg	5 (12.5)		
Chemotherapy group		40 (100)	
Monotherapy		37 (92.5)	
Nabpaclitaxel/docetaxel		14 (35.0)	
Capecitabine		12 (30.0)	
Vinorelbine		5 (12.5)	
Pegylated Liposomal doxorubicin		3 (7.5)	
Etoposide		3 (7.5)	
Combination therapy		3 (7.5)	

Abbreviations: CDK4/6, cyclin‐dependent kinase 4 and 6; CR, complete response; DCR, disease control rate; ECOG PS, Eastern Cooperative Oncology Group performance status; ER, estrogen receptor; OFS, ovarian function suppression; ORR, objective response; PFS, progression‐free survival; PgR, progesterone receptor; PR, partial response; SD, stable disease.

^a^ Statistically significant.

In the abemaciclib group, all patients were female, with 13 (32.5%) premenopausal and 27 (67.5%) postmenopausal. And, 30 patients (75.0%) had an ECOG PS 0. The most common sites of metastasis were bone (82.5%), followed by lymph nodes/soft issues (62.5%), lung (55.0%), and liver (45.0%). In total, 31 patients (77.5%) had visceral disease, and 29 patients (72.5%) had ≥3 metastatic sites. Patients had received a median of three (range: 1–18) prior lines of systemic therapy in the metastatic setting, with a median of two (range: 1–6) prior lines of ET and a median of one prior line of chemotherapy (range: 0–12). In the sequential arm (*n* = 23), patients had received a median of two (range: 1–7) prior lines of systemic therapy, significantly less than that of 6 (range, 3–18) in the nonsequential arm (*n* = 17) (*p* < 0.001). Only eight patients (20.0%) received palbociclib in combination with ET as the first‐line therapy, while the majority of patients (80.0%) received palbociclib plus ET as ≥2nd‐line therapy. Twelve patients (30.0%) did not receive any chemotherapy in the metastatic setting. The median PFS of prior palbociclib in combination with ET was 10 months (range: 2–24 months). The median PFS of prior palbociclib in the sequential arm was 12.0 months significantly longer than 10.0 months in the nonsequential arm (*p* = 0.021). Only 19 patients (47.5%) achieved ≥12 months of PFS for prior palbociclib‐based therapy.

No significant differences were found between the abemaciclib group and the chemotherapy group in age at the start of abemaciclib or chemotherapy, original stage at diagnosis, DFI, and site of metastatic lesion (visceral or nonvisceral), and number of metastatic sites at the start of therapy (*p* >0.05). Compared with the chemotherapy group, the prior palbociclib‐based therapy showed a significantly higher ORR rate in the abemaciclib group (30.0% vs. 5.0%, *p* = 0.003). The median duration of prior palbociclib‐based therapy was significantly longer in the abemaciclib group than in the chemotherapy group (10.0 months vs. 7.0 months, *p* = 0.014). And, patients in the abemaciclib group had received more lines of chemotherapy and systemic therapy than patients in the chemotherapy group (*p* = 0.011 and *p* = 0.026).

### 3.2 Treatment

Among the 40 patients included in the abemaciclib group in this study, 23 (57.5%) patients received sequential abemaciclib plus ET in seven patients with prior palbociclib as the first‐line therapy, while 17 (42.5%) patients received nonsequential abemaciclib plus ET. 45.0% (*n* = 18) initiated abemaciclib at an index dose of 150 mg, 42.5% (*n* = 17) initiated at an index dose of 100 mg, and 12.5% (*n* = 5) at an index dose of 50 mg. Overall, 45.0% of the patients received abemaciclib combined with fulvestrant, 40.0% used abemaciclib with AI, and 15.0% were treated with abemaciclib plus megestrol acetate. All premenopausal patients were concomitantly administered with ovarian function suppression. (Table 1). At the data cutoff on April 30th, 2023, 34 (85.0%) patients had discontinued abemaciclib, primarily due to disease progression (75.0%, *n* = 30), or AEs (5.0%, *n* = 2).

In the chemotherapy group, 37 (92.5%) patients received monotherapy, which was most often nabpaclitaxel/docetaxel (35.0%), capecitabine (30.0%), or vinorelbine (12.5%) (Table 1). At the data cutoff on April 30th, 2023, 39 (97.5%) patients had discontinued chemotherapy, primarily due to disease progression (72.5%, *n* = 29). Only three (7.5%) patients discontinued chemotherapy due to AEs in the control group.

### 3.3 Treatment efficacy

**FIGURE 2:**
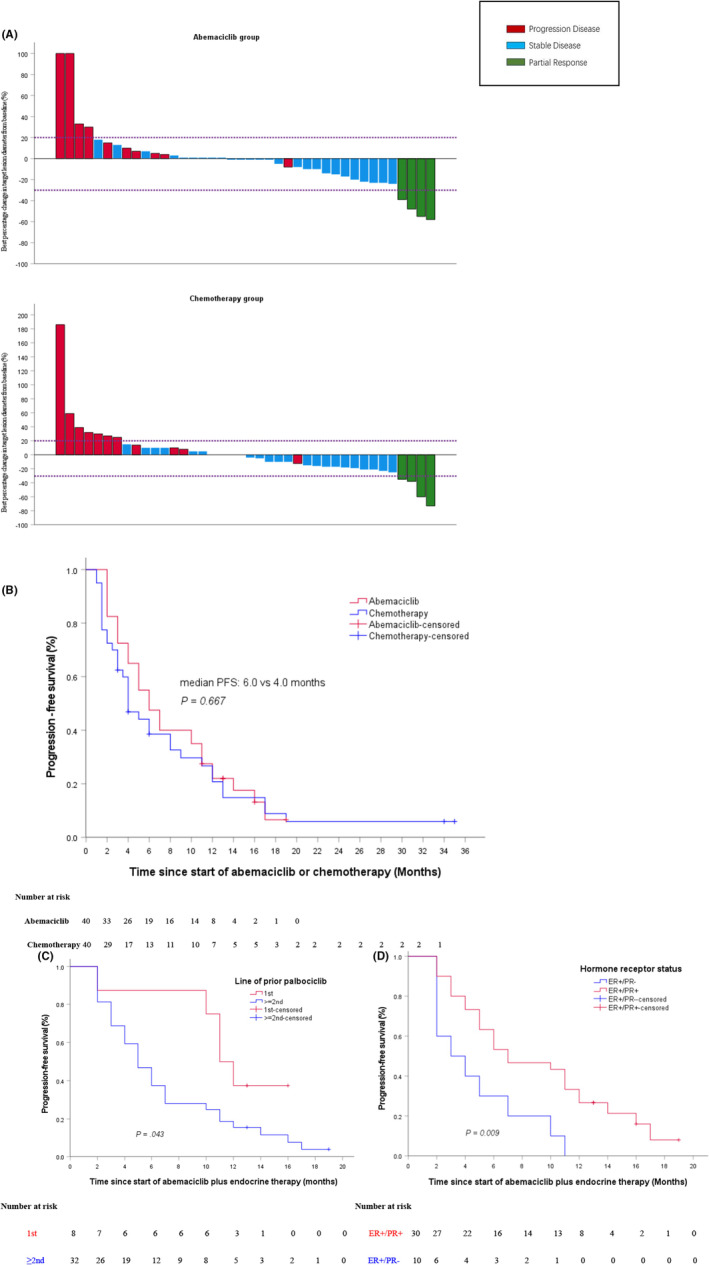
Efficacy evaluation. (A)Waterfall plot of best percentage change in target tumor lesion diameters from baseline in the abemaciclib and chemotherapy groups. (B) Kaplan–Meier curve of progression‐free survival for patients receiving abemaciclib plus endocrine therapy or chemotherapy after progression on palbociclib‐based therapy. (C) Kaplan–Meier curve of progression‐free survival for patients with prior palbociclib as the first line therapy in the metastatic setting vs. patients with prior palbociclib as ≥2nd line therapy in the abemaciclib group. (D) Kaplan–Meier curve of progression‐free survival for ER+/PR+ versus ER+/PR‐ in the abemaciclib group. CI, confidence interval; PFS: progression‐free survival.

All patients were evaluated for efficacy (Table 2).In the abemaciclib group, no patient (0.0%) achieved CR, four (10.0%) patients had a PR, and 26 (65.0%) patients achieved SD (Figure 2A). In the chemotherapy group, no patient (0.0%) achieved CR, 4 (10.0%) had a PR, and 25 (62.5%) patients achieved SD (Figure 2A). The overall ORR was 10.0% (95% CI, 0.2.5–20.0) for patients in the abemaciclib group and 10.0% (95% CI, 2.5–20.0) for patients in the chemotherapy group (*p* = 1.000), DCR was 75.0% (95% CI, 60.0–87.5) in the abemaciclib group and 67.5% (95% CI, 52.5–80.0) in the control group (*p* = 0.622), and CBR of 55.0% (95% CI, 40.0–70.0) in the abemaciclib group and 40.0% (95% CI, 25.0–75.0) in the chemotherapy group (*p* = 0.263), respectively. Furthermore, no significant differences were found between different subgroups in ORR, DCR, or CBR, including between the sequential and nonsequential abemaciclib subgroups. However, the CBR of patients with prior palbociclib as the first‐line therapy in the metastatic setting was numerically higher than those with prior palbociclib as ≥2nd‐line therapy in the abemaciclib group (87% vs. 46.9%, *p* = 0.054).

**TABLE 2:** Summary of treatment efficacy.

Patients	Best objective response *n* (%)				ORR (%) (95% CI)	DCR (%) (95% CI)	CBR (%) (95% CI)	mPFS (months) (95%CI)
	CR	PR	SD	PD				
All patient (*n* = 80)								
Abemaciclib group (*n* = 40)	0 (0.0)	4 (10.0)	26 (65.0)	10 (25.0)	10.0 (2.5–20.0)	75.0 (60.0–87.5)	55.0 (40.0–70.0)	6.0 (3.94–8.06)
Chemotherapy group (*n* = 40)	0 (0.0)	4 (10.0)	25 (62.5)	11 (27.5)	10.0 (2.5–20.0)	67.5 (52.5–80.0)	40.0 (25.0–75.0)	4.0 (2.52–5.49)
*p*					1.000	0.622	0.263	0.667
Abemaciclib plus endocrine therapy								
Line of prior palbociclib								
First line (*n* = 8)	0 (0.0)	1 (12.5)	6 (75.0)	1 (12.5)	12.5 (0.0–37.5)	87.5 (62.5–100.0)	87.5 (62.5–100.0)	11.0 (9.15–12.85)
≥2nd line (*n* = 32)	0 (0.0)	3 (9.4)	20 (62.5)	9 (28.1)	9.4 (0.0–21.9)	71.9 (56.3–84.4)	46.9 (31.3–65.6)	5.0 (3.42–6.58)
*p*					1.000	0.653	0.054	0.043^a^
CDK4/6 inhibitor sequence								
Sequential (n = 23)	0 (0.0)	3 (13.0)	14 (60.9)	6 (26.1)	3 (13.0)	17 (73.9)	12 (52.2)	6.0 (0.00–13.04)
Nonsequential(n = 17)	0 (0.0)	1 (5.9)	12 (70.6)	4 (23.5)	1 (5.9)	13 (76.5)	10 (58.8)	6.0 (4.39–7.61)
*p*					0.624	1.000	0.676	0.369
Abemaciclib‐based regimen								
AI (*n* = 16)	0 (0.0)	3 (18.8)	10 (62.5)	3 (18.8)	3 (18.8)	13 (81.3)	10 (62.5)	7.0 (1.77–12.23)
Fulvestrant (*n* = 18)	0 (0.0)	1 (5.6)	12 (66.7)	5 (27.8)	1 (5.6)	13 (72.2)	8 (44.4)	5.0 (3.62–6.38)
Megestrol acetate (*n* = 6)	0 (0.0)	0 (0.0)	4 (66.7)	2 (33.3)	0 (0.0)	4 (66.7)	4 (66.7)	6.0 (1.47–12.53)
*p*					0.298	0.730	0.471	0.836
Chemotherapy								
Line of prior palbociclib								
First line (*n* = 12)	0 (0.0)	1 (8.3)	8 (66.7)	3 (25.0)	8.3 (0.0–25.0)	75.0 (50.0–100.0)	33.3 (8.3–58.3)	4.0 (2.89–5.11)
≥2nd line (*n* = 28)	0 (0.0)	3 (10.7)	17 (60.7)	8 (28.6)	10.7 (0.0–25.0)	64.3 (46.4–82.1)	42.9 (25.0–60.7)	4.0 (0.00–5.49)
*p*					1.000	0.716	0.729	0.663

Abbreviations: AI, aromatase inhibitor; CBR, clinical benefit rate, defined as complete response + partial response + stable disease ≥6 months; CI, confidence interval; CR, complete response; DCR, disease control rate defined as complete response or partial response or stable disease; mPFS, median progression‐free survival; ORR, objective response rate, defined as complete response, partial response; PR, partial response; SD, stable disease.

^a^ Statistically significant.

The median follow‐up duration was 15.0 months (range: 2.0–28.0 months) in the abemaciclib group and 13.0 months (range: 3.0–51 months) in the chemotherapy group. The median PFS was 6.0 months (95% CI, 3.94–8.06) among patients treated with abemaciclib plus endocrine in the abemaciclib group and 4.0 months (95% CI, 2.52–5.49) among patients treated with chemotherapy in the chemotherapy group (*p* = 0.667), (Figure 2B). Notably, those patients with prior palbociclib as the first‐line therapy in the metastatic setting had significantly longer PFS compared with those with prior palbociclib as ≥2nd line therapy (median PFS: 11.0 vs. 5.0 months, log‐rank *p* = 0.043, Figure 2C). Similarly, patients with ER+/PR+ tumors had significantly longer PFS compared to patients with ER+/PR‐ tumors (median PFS: 7.0 vs. 3.0 months, log‐rank *p* = 0.009, Figure 2D). Median PFS in the subgroup without prior chemotherapy in the metastatic setting was 12.0 months (95% CI, 10.33–13.67), as compared to 5.0 months (95% CI, 3.52–6.48) in the subgroup with ≥1 prior chemotherapy in the metastatic setting, with log‐rank *p*‐ value of 0.004. Among patients with prior palbociclib as first‐line therapy, the median PFS of abemaciclib plus ET was 12.0 months (95% CI, 9.43–14.57) in the sequential arm. Nonetheless, those patients who had ≥12 months of PFS during prior palbociclib‐based therapy only had a numerical trend towards PFS improvement compared with those patients who had less than 12 months of PFS during prior palbociclib‐based therapy (median PFS: 7.0 vs. 5.0 months, log‐rank *p* = 0.931). There was no difference in PFS between the sequential abemaciclib subgroup and the nonsequential abemaciclib subgroup (median PFS: 6.0 vs. 6.0 months, log‐rank *p* = 0.308). And, the median PFS was 7.0 months (95% CI, 1.77–12.23) for patients receiving abemaciclib plus AI (*n* = 16), 5.0 months (95% CI, 3.62–6.38) for patients receiving abemaciclib plus fulvestrant (*n* = 18), and 6.0 months (95% CI, 1.47–10.53) for patients with abemaciclib plus megestrol acetate (*n* = 6) (log‐rank *p* = 0.836).

**FIGURE 3:**
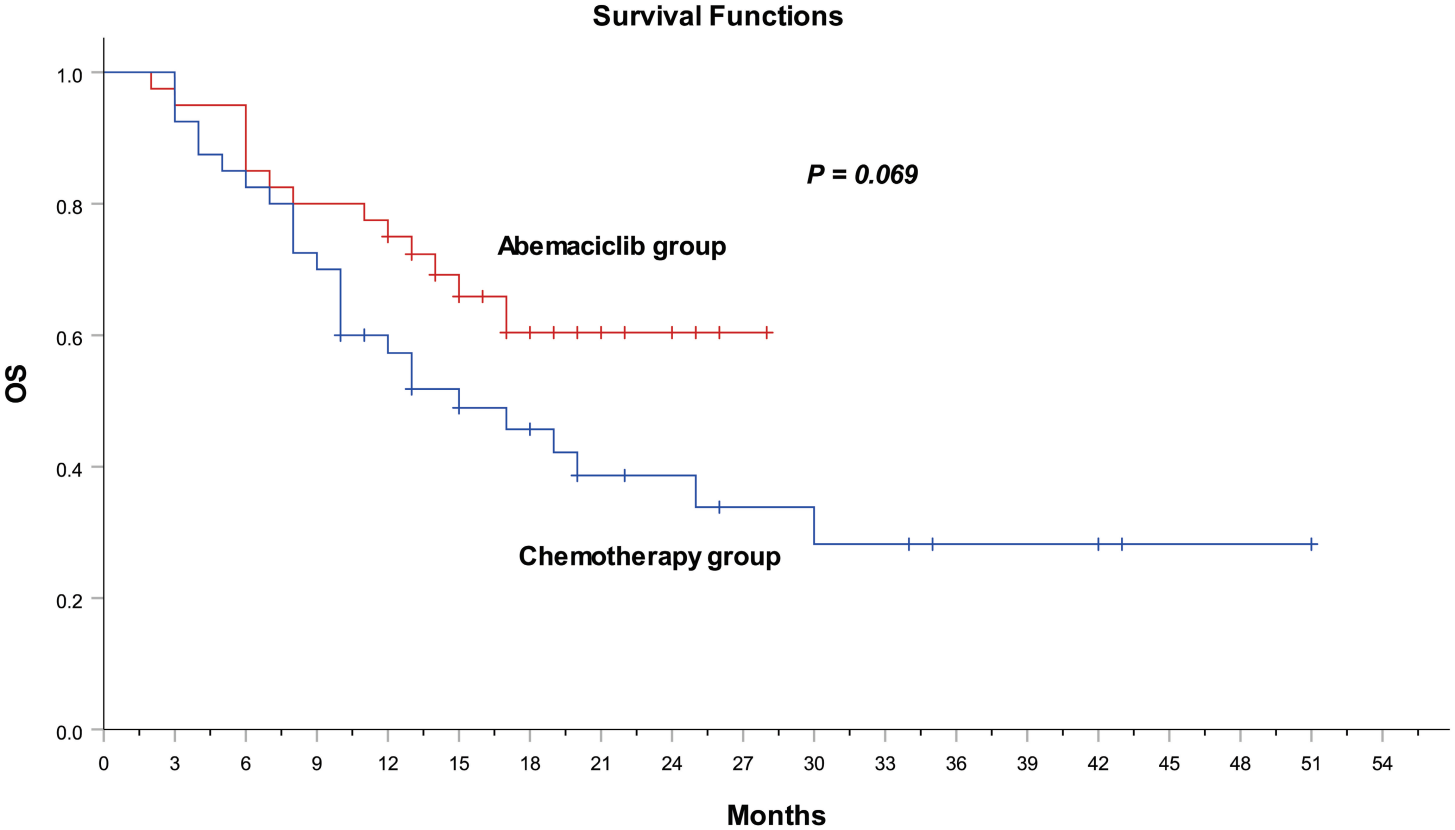
Kaplan–Meier analysis of overall survival in the abemaciclib and chemotherapy groups. OS, overall survival.

The overall OS rate at 12 months was 77.5% (95% CI, 64.0–91.0) in the abemaciclib group and 57.5% (95% CI, 40.0–72.5) in the chemotherapy group. The median OS was not reached in patients who received abemaciclib plus endocrine therapy due to 65% of patients being alive at the data cut off time and was 15.0 months (95% CI, 7.68–22.3) in patients who received chemotherapy. However, there was no significant difference observed in OS between the abemaciclib and chemotherapy groups (log‐rank *p* = 0.069, Figure 3).

### 3.4 Clinical factors associated with PFS in the abemaciclib group

The univariate factors associated with PFS in the abemaciclib group are shown in Table 3. The median PFS was significantly longer in patients who were diagnosed with ER+/PR+ tumor, who had received prior palbociclib as the first‐line therapy, or who received no prior chemotherapy in the metastatic setting. Meanwhile, the median PFS was shorter in patients with liver metastasis, patients with visceral metastasis, or patients with more than three metastatic sites. However, the multivariable Cox regression analysis showed only ER+/PR+ was an independent factor associated with a longer PFS (HR: 0.435 [95% CI, 0.195–0.967], *p* = 0.041) (Table 3).

**TABLE 3:** Univariate and multivariate Cox regression analysis of factors associated with progress‐free survival in the abemaciclib group.

Variable	Univariate analysis			Multivariate analysis		
	HR	95% CI	*p* value	HR	95% CI	*p* value
Age						
<60	1					
≥60	0.891	0.452–1.757	0.739	‐	‐	‐
Menopausal status						
Premenopausal	1					
Postmenopausal	0.850	0.412–1.753	0.660	‐	‐	‐
ECOG PS status						
1	1					
0	0.533	0.252–1.128	0.100	‐	‐	‐
Disease‐free survival						
>2 years	1					
≤2 years	0.369	0.049–2.786	0.334	‐	‐	‐
De novo	0.921	0.452–1.877	0.820	‐	‐	‐
Hormone receptor status						
ER positive/PgR negative	1					
ER positive/PgR positive	0.392	0.181–0.849	0.018	0.435	0.195–0.967	0.041
Liver metastasis						
Yes	1					
No	0.406	0.200–0.825	0.013	0.776	0.340–1.770	0.546
Visceral metastasis						
Yes	1					
No	0.360	0.137–0.945	0.038	0.753	0.231–2.453	0.638
Number of metastatic sites						
≥3	1					
<3	0.392	0.192–0.800	0.010	0.638	0.278–1.462	0.288
Line of prior endocrine therapy in the metastatic setting						
≥2	1					
1	0.619	0.279–1.375	0.239	‐	‐	‐
Line of prior chemotherapy in the metastatic setting						
≥1	1					
0	0.320	0.135–0.754	0.009	0.563	0.163–1.937	0.362
CDK4/6 inhibitor sequence						
Nonsequential	1					
Sequential	0.680	0.340–1.363	0.277	‐	‐	‐
Line of prior palbociclib therapy						
≥2nd	1					
1st	0.405	0.155–1.059	0.065	0.796	0.222–2.854	0.726
Median PFS of prior pablcociclib						
<12 months	1					
≥12 months	0.864	0.440–1.698	0.672	‐	‐	‐
Abemaciclib‐based regimen						
Megestrol Acetate	1					
Aromatase inhibitor	0.744	0.264–2.095	0.575	‐	‐	‐
Fulvestrant	0.809	0.291–2.247	0.685	‐	‐	‐

Abbreviations: CDK4/6, cyclin‐dependent kinase 4 and 6; CI, confidence interval; ECOG PS, Eastern Cooperative Oncology Group performance status; ER, estrogen receptor; HR, hazard ratio; PFS, progression‐free survival; PgR, progesterone receptor.

### 3.5 Safety

There was no significant difference in dose modification between abemaciclib and chemotherapy (*p* = 0.813). In the abemaciclib group, dose reduction of abemaciclib occurred in 11 patients (27.5%). Neutropenia was the most comm reason for dose reduction (*n* = 5; 12.5%), followed by diarrhea (*n* = 3; 7.5%). Only two patients (5.0%) discontinued abemaciclib due to grade 2 pneumonitis.

In total, AEs of any grade were reported in 35 (87.5%) patients in the abemaciclib group and 32 (80.0%) in the chemotherapy group. Furthermore, AEs of grade 3/4 were documented in eight patients (20.0%) who received abemaciclib, and in 11 (27.5%) who received chemotherapy, respectively (Table 4). No significant differences were observed in AEs of any grade or grade 3/4 between the two groups (all *p* > 0.05).

**TABLE 4:** Incidence of adverse events in the abemaciclib and chemotherapy groups (≥5% or any toxicities with grade 3 or 4).

Adverse events	Grade 1 *n* (%)		Grade 2 *n* (%)		Grade 3 *n* (%)		Grade 4 *n* (%)		Any Grade *n* (%)		Grade 3/4 *n* (%)	
	Abema	Chemo	Abema	Chemo	Abema	Chemo	Abema	Chemo	Abema	Chemo	Abema	Chemo
Patients with any event	30 (75.0)	28 (70,0)	23 (57.5)	18 (45.0)	8 (20.0)	10 (25.0)	2 (5.0)	4 (10.0)	35 (87.5)	32 (80.0)	8 (20.0)	11 (27.5)
Hematologic												
Leukopenia	17 (42.5)	4 (10.0)	12 (30.0)	8 (20.0)	2 (5.0)	5 (12.5)	0 (0.0)	2 (5.0)	31 (77.5)	19 (47.5)	2 (5.0)	7 (17.5)
Neutropenia	5 (12.5)	1 (2.5)	12 (30.0)	6 (15.0)	4 (10.0)	4 (10.0)	1 (2.5)	4 (10.0)	22 (55.0)	15 (37.5)	5 (12.5)	8 (20.0)
Anemia	6 (15.0)	14 (35.0)	6 (15.0)	4 (10.0)	1 (2.5)	1 (2.5)	0 (0.0)	0 (0.0)	13 (32.5)	19 (47.5)	1 (2.5)	5 (12.5)
Thrombocytopenia	1 (2.5)	2 (5.0)	3 (7.5)	3 (7.5)	1 (2.5)	0 (0.0)	0 (0.0)	0 (0.0)	5 (12.5)	5 (12.5)	1 (2.5)	0 (0.0)
Nonhematologic												
Nausea	0 (0.0)	1 (2.5)	2 (5.0)	1 (2.5)	0 (0.0)	0 (0.0)	0 (0.0)	0 (0.0)	2 (5.0)	2 (5.0)	0 (0.0)	0 (0.0)
Vomiting	0 (0.0)	0 (0.0)	0 (0.0)	0 (0.0)	0 (0.0)	1 (2.5)	0 (0.0)	0 (0.0)	0 (0.0)	1 (2.5)	0 (0.0)	1 (2.5)
Diarrhea	7 (17.5)	0 (0.0)	4(10.0)	0 (0.0)	0 (0.0)	1 (2.5)	0 (0.0)	0 (0.0)	11 (27.5)	1 (2.5)	0 (0.0)	1 (2.5)
Constipation	0 (0.0)	2 (5.0)	0 (0.0)	0 (0.0)	0 (0.0)	0 (0.0)	0 (0.0)	0 (0.0)	0 (0.0)	2 (5.0)	0 (0.0)	0 (0.0)
ALT increased	6(15.0)	10 (25.0)	1 (2.5)	1 (2.5)	1 (2.5)	0 (0.0)	0 (0.0)	0 (0.0)	8 (20.0)	11 (27.5)	1 (2.5)	0 (0.0)
AST increased	6(15.0)	7 (17.5)	3 (7.5)	2 (5.0)	1 (2.5)	1 (2.5)	0 (0.0)	0 (0.0)	10 (25.0)	10 (25.0)	1 (2.5)	1 (2.5)
GGT increased	3 (7.5)	1 (2.5)	1 (2.5)	0 (0.0)	1 (2.5)	0 (0.0)	1 (2.5)	0 (0.0)	6 (15.0)	1 (2.5)	2 (5.0)	0 (0.0)
Hypertriglyceridemia	8 (20.0)	2 (5.0)	2 (5.0)	0 (0.0)	1 (2.5)	0 (0.0)	0 (0.0)	0 (0.0)	11 (27.5)	2 (5.0)	1 (2.5)	0 (0.0)
Cholesterol high	7 (17.5)	1 (2.5)	2 (5.0)	0 (0.0)	0 (0.0)	0 (0.0)	0 (0.0)	0 (0.0)	9 (22.5)	1 (2.5)	0 (0.0)	0 (0.0)
Bilirubin increased	1 (2.5)	7 (17.5)	1 (2.5)	2 (5.0)	1 (2.5)	1 (2.5)	1 (2.5)	0 (0.0)	4 (10.0)	10 (25.0)	2 (5.0)	1 (2.5)
Fatigue	3 (7.5)	4 (10.0)	0 (0.0)	1 (2.5)	0 (0.0)	0 (0.0)	0 (0.0)	0 (0.0)	3 (7.5)	5 (12.5)	0 (0.0)	0 (0.0)
Rash	3 (7.5)	1 (2.5)	0 (0.0)	0 (0.0)	0 (0.0)	0 (0.0)	0 (0.0)	0 (0.0)	3 (7.5)	1 (2.5)	0 (0.0)	0 (0.0)
Anorexia	0 (0.0)	0 (0.0)	2 (5.0)	1 (2.5)	0 (0.0)	0 (0.0)	0 (0.0)	0 (0.0)	2 (5.0)	1 (2.5)	0 (0.0)	0 (0.0)
Pneumonitis	0 (0.0)	0 (0.0)	2 (5.0)	0 (0.0)	0 (0.0)	0 (0.0)	0 (0.0)	0 (0.0)	2 (5.0)	0 (0.0)	0 (0.0)	0 (0.0)
PPE	0 (0.0)	5 (12.5)	0 (0.0)	0 (0.0)	0 (0.0)	0 (0.0)	0 (0.0)	0 (0.0)	0 (0.0)	5 (12.5)	0 (0.0)	0 (0.0)
Neurosensory	0 (0.0)	5 (12.5)	0 (0.0)	0 (0.0)	0 (0.0)	1 (2.5)	0 (0.0)	0 (0.0)	0 (0.0)	6 (15.0)	0 (0.0)	1 (2.5)
Hyperglycemia	0 (0.0)	4 (10.0)	0 (0.0)	0 (0.0)	0 (0.0)	0 (0.0)	0 (0.0)	0 (0.0)	0 (0.0)	4 (10.0)	0 (0.0)	0 (0.0)

Abbreviations: Abema, abemaciclib; ALT, alanine aminotransferase; AST, aspartate aminotransferase; Chemo, chemotherapy; GGT, γ‐glutamyltransferase; PPE, Palmar‐plantar‐erythrodysaesthesia.

In the abemaciclib group, the most common AEs were leukopenia (n = 31; 77.5%), neutropenia (*n* = 25; 55.0%), anemia (*n* = 13; 32.5%), diarrhea (*n* = 11; 27.5%), and hypertriglyceridemia (*n* = 11; 27.5%). AEs of grade 3/4 were reported in 8 (20.0%) patients. The most frequent grade 3/4 AEs included neutropenia (12.5%), leukopenia (5.0%), bilirubin increased (5.0%), and γ‐glutamyltransferase (GGT) (5.0%). No patient experienced fatal AEs.

## 4 DISCUSSION

The optimal therapeutic paradigm after progression on a CDK4/6 inhibitor becomes an unmet clinical need. One of the potential strategies is sequential CDK4/6 inhibitor after disease progression with CDK4/6 inhibitor‐based therapy. To date, three randomized phase II trials have been reported to evaluate this approach.^39^, ^40^, ^41^ Only the MAINTAIN trial demonstrated positive results,^39^ whereas the PACE trial and PALMIRA trial showed negative results.^40^, ^41^ Therefore, more data are needed to determine the sequence and principle of reuse of CDK4/6 inhibitor.

In this retrospective study, we found that abemaciclib plus different ET achieved the median PFS of 6.0 months, numerically longer than the median PFS of 4.0 months from chemotherapy after progression on prior palbociclib in combination with ET in Chinese patients with HR+/HER2‐ MBC, with a well tolerable safety profile. Furthermore, our findings showed ER+/PR+ subtype was an independent factor associated with a longer PFS.

A multicenter respective study by Li Y, et al analyzed the clinical outcome of subsequent chemotherapy (*n* = 147) or endocrine therapy (*n* = 53) after progression on palbociclib in HR+/HER2‐ MBC in five Chinese institutions.^42^ Results showed the median PFS was 5.6 months in the chemotherapy group and 4.6 months in the endocrine therapy group. The endocrine therapy regimens included ET alone (*n* = 7), chidamide plus ET (*n* = 21), and everolimus plus ET (*n* = 15). Of note, no patient received abemaciclib plus ET after progression on palbociclib in that study. Besides, Abemaciclib monotherapy showed active antitumor efficacy in the heavily pretreated patients with refractory HR+/HER2‐MBC.^30^, ^43^, ^44^ However, all patients did not receive prior any CDK4/6 inhibitor in those trials.^30^, ^43^, ^44^ Wander et al reported the first multicenter retrospective study to evaluate the efficacy and safety of abemaciclib after progression on prior palbociclib plus ET in patients with HR+/HER2‐ MBC in 2021.^34^ In that study, the median PFS was 5.3 months and the median OS was 17.2 months. Our study showed the median PFS was 6.0 months and the 12‐month OS rate was 77.5% in patients who received abemaciclib plus different ET after progression on palbociclib in the abemaciclib group, numerically longer than the results from Wander et al. These numerical differences might be due to that 80.5% of patients received abemaciclib plus ET (25.3% the same ET) and 19.5% of patients received abemaciclib monotherapy in that study,^34^ whereas, 100% of patients received abemaciclib plus different ET in our study. Of note, a recent study showed a median PFS of 6.1 months in 52 patients who received abemaciclib monotherapy (28.8%) or plus ET (71.2%) after progression on prior palbociclib. Importantly, this study found palbociclib‐resistant cells upregulated G2‐M pathways and abemaciclib could mediate G2 arrest of palbociclib‐resistant cells. Furthermore, these findings were confirmed by both patient‐derived xenografts (PDX) and PDX‐derived organoids models of palbociclib‐resistant BC.^45^ Overall, these promising results indicate that the use of abemaciclib plus different ET after progression on prior palbociclib might be a feasible treatment strategy in addition to chemotherapy. However, MAINTAIN trial offered the initial evidence that using ribociclib and switching ET had significantly improved the median PFS compared to placebo plus switching ET after progression on a prior CDK4/6 inhibitor (86.5% palbociclib) (5.29 months vs. 2.76 months).^39^ Whereas, continuing palbociclib plus switching ET after progression on palbociclib did not show clinical benefit in the phase II PACE trial^40^ and PALMIRA trial.^41^ These inconsistent findings imply that the rechallenge strategy of CDK4/6 inhibitors might not be suitable for all CDK4/6 inhibitors.

Our results showed that patients with prior palbociclib as the first‐line therapy had a significantly longer median PFS versus patients with prior palbociclib as ≥2nd‐line therapy (11.0 vs. 5.0 months, *p* = 0.043), generally similar to that observed in an analysis of 1170 patients' subsequent therapy after palbociclib in Japan by Sawaki et al (10.9 months).^46^ Interestingly, patients with no prior chemotherapy in the metastatic setting had a significantly longer PFS than patients with ≥1 prior chemotherapy (12.0 vs. 5.0 months, *p* = 0.004). However, We did not observe sequential abemaciclib obtained longer PFS compared to nonsequential abemaciclib (6.0 vs. 6.0 months) though patients received fewer lines of prior systemic therapy and had longer PFS from prior palbociclib in the sequential arm compared to the nonsequential arm, in line with the study by Navarro‐Yepes et al (6.2 vs. 6.1 months),^45^ contrary to the retrospective study by Wander et al, (8.4 vs. 3.0 months, *p* = 0.0013).^47^ Interestingly, Navarro‐Yepes et al found sequential abemaciclib obtained significantly longer median OS for patients treated sequentially (42.7 months vs.17.3 months).^45^ Real‐world data from the Flatiron Health Network showed that continuation of CDK4/6 inhibitor after progression on first‐line CDK4/6 inhibitor was significantly associated with improved PFS and OS.^48^ Hence, sequential abemaciclib plus switching ET after progression on first‐line palbociclib warrants further investigation in randomized prospective trials. Estrogen receptor 1 (*ESR1*) mutations are one of mechanisms for acquired endocrine therapy resistance.^49^, ^50^, ^51^ Previous studies showed that fulvestrant is still effective in patients with *ESR1* mutant BC.^52^, ^53^ We hypothesize that fulvestrant might be the optional ET partner with abemaciclib after palbociclib plus AI. Therefore, all patients treated with abemaciclib plus fulvestrant had previously received an AI or AI plus palbociclib and developed disease progression in our study. Conversely, all patients treated with abemaciclib plus AI had previously received fulvestrant or fulvestrant plus palbociclib and developed disease progression afterward. Interestingly, our results showed patients treated with abemaciclib plus AI had a slightly numerically longer median PFS compared with patients with abemaciclib plus fulvestrant (7.0 months vs. 5.0 months), partly possibly due to the small sample size and very heterogeneous population (1–18 prior lines of systemic therapy). In the present study, ER+/PR+ tumors had significantly longer PFS versus ER+/PR‐ tumors (7.0 vs. 3.0 months, P = 0.009). Furthermore, multivariable Cox regression analysis showed only ER+/PR+ was associated with a longer PFS of abemaciclib plus switching ET after progression on prior palbociclib. So far, no similar results have been reported. However, previous studies showed that ER+/PR+ early BC had a better prognosis and a better benefit from adjuvant endocrine therapy compared with ER+/PR‐ tumor.^54^, ^55^, ^56^ Therefore, patients with ER+/PR+ MBC might be more suitable for abemaciclib after prior palbociclib progression than ER+/PR‐ MBC. Further investigation is warranted.

Our study showed that only 5.0% of patients discontinued abemaciclib due to AEs, while 27.5% of patients experienced dose reduction of abemaciclib, less frequent vs. the MONARCH 2^13^ and MONARCH 3^12^ trials. Although 87.5% of patients experienced AEs, the majority of AEs were grade 1 or grade 2 in our study. The incidence and severity of AEs are lower than previously reported.^12^, ^13^ These findings may be likely due to only 45% of patients initiating abemaciclib at the standard recommended prescribing dose based on physicians' decisions according to patients' condition in our study. Rugo et al reported dose reduction of abemaciclib did not impair the PFS benefit of abemaciclib through analyses from the MONARCH 2 and MONARCH 3 trials.^57^ Therefore, our mode of dose adjustment might improve the therapeutic compliance of heavily pretreated patients without impairing the efficacy of abemaciclib.

There are several limitations to our study. First, this study was a retrospective study conducted in a single center. The outcomes from real‐world settings might be influenced by confounding factors, such as concomitant diseases and medications. Second, the small sample size and very heterogeneous population (received 1–18 prior lines of systemic therapy) also limited the extent of analyses and potential generalizability of these results. Especially, the analyses were not limited only to those patients with no prior lines of systemic therapy. Third, the OS data was not mature due to the short follow‐up. Finally, the intrinsic limitations of the retrospective nature, the lack of blinded response assessment at predefined intervals, and physician subjectivity in terms of the treatment regimen. Therefore, these findings from this study should be interpreted with caution.

In conclusion, we evaluated the efficacy and safety of abemaciclib plus switching ET versus chemotherapy after progression on prior palbociclib‐based ET in patients with HR+/HER2‐ MBC in the single‐center retrospective study. We found that abemaciclib plus switching ET might be one of optional treatment strategies for patients with HR+/HER2‐ MBC after progression on prior palbociclib in addition to chemotherapy.

## AUTHOR CONTRIBUTIONS

**Hanfang Jiang:** Conceptualization (equal); data curation (equal); formal analysis (equal); funding acquisition (lead); investigation (equal); methodology (equal); project administration (equal); resources (equal); software (equal); supervision (equal); validation (equal); writing – original draft (lead); writing – review and editing (equal). **Jianxin Zhong:** Data curation (equal); formal analysis (equal); investigation (equal); methodology (equal); software (equal); validation (equal); visualization (equal); writing – review and editing (equal). **Jing Wang:** Data curation (equal); investigation (equal); validation (equal); visualization (equal); writing – review and editing (equal). **Guohong Song:** Investigation (equal); resources (equal); validation (equal); writing – review and editing (equal). **Lijun Di:** Investigation (equal); resources (equal); validation (equal); writing – review and editing (equal). **Bin Shao:** Investigation (equal); resources (equal); validation (equal); writing – review and editing (equal). **Ruyan Zhang:** Investigation (equal); resources (equal); validation (equal); writing – review and editing (equal). **Yaxin Liu:** Investigation (equal); validation (equal); writing – review and editing (equal). **Anjie Zhu:** Investigation (equal); resources (equal); validation (equal); writing – review and editing (equal). **Nan Wang:** Investigation (equal); validation (equal); writing – review and editing (equal). **Huiping Li:** Conceptualization (equal); data curation (equal); investigation (equal); methodology (equal); project administration (equal); resources (equal); supervision (equal); validation (equal); writing – original draft (equal); writing – review and editing (equal).

## FUNDING INFORMATION

This work was supported by Beijing Medical Award Foundation (grant number: YXJL‐2018‐0067‐0080).

## CONFLICT OF INTEREST STATEMENT

The authors declare no conflict of interest.

## ETHICS STATEMENT

The study was conducted in accordance with the Declaration of Helsinki (as revised in 2013). The study was approved by the Ethics Committee of Peking University Cancer Hospital (Approval ID: 2022KT27). Patient consent was waived due to the observational, retrospective nature of this study.

## Data Availability

The data that support the findings of our study are available on request from the corresponding authors.
